# Ambient Temperature Self-Blowing Tannin-Humins Biofoams

**DOI:** 10.3390/polym12112732

**Published:** 2020-11-17

**Authors:** Xinyi Chen, Nathanael Guigo, Antonio Pizzi, Nicolas Sbirrazzuoli, Bin Li, Emmanuel Fredon, Christine Gerardin

**Affiliations:** 1 LERMAB, University of Lorraine, 27 rue Philippe Seguin, 88000 Epinal, France; xinyi.chen@univ-lorraine.fr (X.C.); emmanuel.fredon@univ-lorraine.fr (E.F.); 2 Department of Chemistry, University of the Cote d’Azur, 06103 Nice, France; Nathanael.GUIGO@univ-cotedazur.fr (N.G.); Nicolas.SBIRRAZZUOLI@univ-cotedazur.fr (N.S.); bin.li@univ-cotedazur.fr (B.L.); 3 LERMAB, University of Lorraine, Boulevard des Aiguillettes, 54000 Nancy, France; christine.gerardin@univ-lorraine.fr

**Keywords:** humins, foams, tannins, tannin–furanic foams, ambient temperature, self-blowing, matrix assisted laser desorption ionization time-of-flight spectrometry (MALDI ToF)

## Abstract

Ambient temperature self-blowing tannin–furanic foams have been prepared by substituting a great part—even a majority—of furfuryl alcohol with humins, a polyfuranic material derived from the acid treatment at high temperature of fructose. Closed-cell foams were prepared at room temperature and curing, while interconnected-cell foams were prepared at 80 °C and curing, this being due to the more vigorous evaporation of the solvent. These foams appear to present similar characteristics as other tannin–furanic foams based only on furfuryl alcohol. A series of tannin–humins–furfuryl alcohol oligomer structures have been defined indicating that all three reagents co-react. Humins appeared to react well with condensed tannins, even higher molecular weight humins species, and even at ambient temperature, but they react slower than furfuryl alcohol. This is due to their high average molecular weight and high viscosity, causing their reaction with other species to be diffusion controlled. Thus, small increases in solvent led to foams with less cracks and open structures. It showed that furfuryl alcohol appears to also have a role as a humins solvent, and not just as a co-reagent and self-polymerization heat generator for foam expansion and hardening. Stress-strain for the different foams showed a higher compressive strength for both the foam with the lowest and the highest proportion of humins, thus in the dominant proportions of either furfuryl alcohol or the humins. Thus, due to their slower reactivity as their proportion increases to a certain critical level, more of them do proportionally participate within the expansion/curing time of the foam to the reaction.

## 1. Introduction

Heat insulation capacity, along with light, weight and sound absorption capacity are today sought-after foam characteristics, as foams are widely used in a number of applications where such properties are essential. Totally biosourced foams have recently been the focus of considerable research. A few types of ambient temperature, or even moderate temperature, self-blowing foams derived by mostly biosourced raw materials have been described in the literature. These have been based either on the self-blowing reaction of tannins with furfuryl alcohol, these being the older types [1,2,3,4,5,6], or on mono-and disaccharides non-isocyanate polyurethanes (NIPU) [7,8,9], or on tannin-based NIPUs [10], or even on tannin–monosaccharide NIPUs [11], or on mixtures of these strategies of approach. The emphasis here is on ambient temperature self-blowing, the use of temperature to obtain foams being less attractive for a certain number and types of applications.

Condensed flavonoid tannins are vegetable polyphenolics, widespread in the plants world and commonly used as a raw material in a number of applications. Among these applications, their use to prepare biosourced foams for thermal and acoustic insulation and other applications has already attracted some attention [2,5,6,12]. Condensed tannins are mainly composed of polyhydroxy-flavan-3-ol and flavan-3,4-diol oligomers linked by carbon–carbon bonds between flavonoid monomer units [1]. The most used approach reported to prepare self-blowing tannin-based foams is by the acid condensation of tannins and furfuryl alcohol [1], with foam expansion driven by a blowing agent activated by the temperature increase caused by the acid self-condensation of furfuryl alcohol [1,2,5,6,13,14,15,16,17,18,19,20].

Humins are dark and viscous residual products obtained by the acid treatment of polysaccharides [21,22]. Humins coming from the acid conversion of fructose or glucose do yield polyfuranic materials still carrying furanic alcohol moieties [21,22,23,24,25]. Thus, under very acid conditions, humins can self-crosslink if heated, and the reaction can be accelerated by adding a strong acid initiator, such as p-toluene sulfonic acid (pTSA) [26]. Humins’ solution in water can be employed, for instance, to create upon thermal treatment a polyfuranic network within wood cells. This so-called ‘humination of wood’ is following the same approach as in furfurylation of wood with aqueous furfuryl alcohol (FA) solutions [27]. Pure humins foams have been prepared by applying heat under very acid conditions [28]. However, this approach yields foams of a very limited variety of characteristics. This is due to the limited possibility of formulation of these heterogeneous distribution materials when foamed alone [28]. It is for this reason that some attempts could be made to mix such waste materials with other materials, in order to formulate foams more adaptable to a greater variety of situations. The only previous attempt of this approach was to react epoxidized linseed oil with humins [29]. Such a system was used to prepare thermoset resins and not strictly used to prepare foams. However, it used the addition of a synthetic mercaptan-terminated hardener that, notwithstanding its general use as a rapid curing agent for synthetic epoxy resins at ambient temperatures, caused the epoxidized linseed oil plus humins mix to need to be cured for several hours at 130 °C.

The goal set in this paper was rather restrictive, namely to prepare an ambient temperature self-blowing biobased foam also using the humins. For these reasons, a condensed tannin extract was chosen for such a purpose, as on top of presenting all the needed characteristics also possesses a very advantageous environmental imprint [30,31]. This paper then deals with the preparation and characterization of ambient temperature self-blowing tannin–furanic totally biosourced rigid foams in which humins represent up to the major proportion of their furanic component.

## 2. Materials and Methods

### 2.1. Materials

Commercial mimosa tannin (Acacia mearnsii, De Wild) bark extract and furfuryl alcohol (FA) were supplied by Silva Chimica (St. Michele Mondovi, Italy). Polyfuranic humins were laboratory prepared by the acid treatment of fructose at the Dept. of Chemistry, Université de la Cote d’Azur, Nice. p-Toluene sulfonic acid (p-TSA) was obtained from Sigma Aldrich (St. Louis, MO, USA). Glyoxal 40% solution (Acros Organics, Geel, Belgium).

### 2.2. Preparation of Humins

The humins were prepared from the acid-catalyzed dehydration of D-fructose in aqueous media following previous experimental protocols [23,24,25]. First, a mixture of 1 M aqueous solution of D-fructose (100 mL) and 0.01 M H_2_SO_4_ was prepared. This mixture was introduced into a 300 mL Teflon lined hydrothermal synthesis autoclave reactor and heated at 150 or 180 °C during 6 h. After reaction a solid residue was isolated by filtration on a 0.2 µm filter and washed several times with about 1 L of deionized water. The solid humins were then dried under vacuum at 80 °C during 16 h and ground into powder. The mass yields (i.e., mass % of humins based on initial fructose basis) was 18 ± 1 % at 150 °C and 30 ± 2 % at 180 °C in good agreement with van Zandvoort et al. [23].

### 2.3. Preparation of Tannin-Humin Foams

Two different series of tannin–humins foams were prepared.

The initial exploratory series was composed of the following formulations:

1 FA + glyoxal control, thus an only furanic foam without tannin [32]
2 FA + glyoxal + humins: 2.5 g humins + 9 g FA + 11.4 g glyoxal + 4 g olive powder + 3.6 g p-TSA+ 1.5 DE (foaming agent); thus, a mixed furfuryl alcohol-humins. An only furanic foam.
3 Tannin + FA + humins (epoxy): 12 g tannin + 5.2 g FA + 4.4 g humins + 6 g p-TSA + 1.5 g DE + 6.6 g water.
4 Tannin + FA + humins: 15 g tannin + 5.2g FA + 3.7 g humins + 6 g p-TSA + 1.5 g DE + 3 g water.

Formulations 1 (control) 2 and 3 were self-blown and cured at ambient temperature (24 °C), while formulation 4 was cured at 80 °C to qualitatively observe the difference in morphology between foam 3 and foam 4. Their differences in composition were used to ensure a stronger cell walls for foam 4, as broken cell walls were expected by curing at 80 °C.

After these initial formulations the systematic study that followed used the formulations shown in Table 1. All the formulations were self-blown at ambient temperature (23 °C).

### 2.4. MALDI-ToF Analysis

Samples for matrix-assisted laser desorption ionization time-of-flight (MALDI-ToF) analysis were prepared first by dissolving 5 mg of sample powder in 10 mL of a 50:50 *v*/*v* acetone/water solution. Then 10 mg of this solution was added to 10 μL of a 2,5-dihydroxy benzoic acid (DHB) matrix to obtain a homogeneous solution. The locations dedicated to the samples on the analysis plaque were first covered with 2 μL of a NaCl solution 0.1 M in 2:1 *v*/*v* methanol/water, and pre-dried. Then, 1 μL of the sample solution was placed on its dedicated location and the plaque was dried again. The reference substance used for the equipment calibration was red phosphorus. MALDI-ToF spectra were obtained using an Axima-Performance mass spectrometer from Shimadzu Biotech (Kratos Analytical Shimadzu Europe Ltd., Manchester, UK) using a linear polarity positive tuning mode. The measurements were carried out making 1000 profiles per sample with two shots accumulated per profile. The spectra precision is of ±1 Da.

For the MALDI ToF analysis the foam sample was ground to a very fine powder homogeneously covered on the target point of dedicated analysis plaque through series of steps before testing. The target testing spots were first covered with the 1.5 μL of a 0.1 M NaCl (in a methanol:water mixture (1:1)) and then completely dried. Secondly, 7.5 mg of samples were dissolved into a 1 mL 50:50 *v*/*v* water/acetone mixture solution. Then, 1.5 μL of this solution was mixed with 1.5 μL of DHB (2,5-dihydroxy benzoic acid) matrix to obtain a homogenous solution. Finally, 1.5 μL of sample-DHB solution were placed on the corresponding testing spots, and dried completely. Before each test, the device was calibrated by using as reference red phosphorous. The measurements were carried out making 1000 profiles per sample with two shots accumulated per profile. The spectra precision is of ±1 Da.

### 2.5. FTIR

Fourier transform infra-red (FTIR) analysis was carried out using a Shimadzu IR Affinity-1 (Shimadzu Europe Ltd., Manchester, UK) spectrophotometer. A blank sample tablet of potassium bromide, ACS reagent from ACROS Organics (Geel, Belgium), was prepared for the reference spectra. Similar tablets were prepared by mixing potassium bromide with 5% by weight of the sample powders. The spectra were plotted in percentage transmittance by combining 32 scans with a resolution of 2.0 cm^−1^ in the 400–4000 cm^−1^ range.

### 2.6. Apparent Density

According to the standard method of ASTM D1622-08, all testing foam samples were prepared to a size of 30 × 30 × 30 mm. The ratio of weight to cubic volume of the specimen volume was defined as density. Five sample repeats were tested for each foam.

### 2.7. Scanning Electron Microscopy (SEM) Analysis

Scanning electron microscopy (SEM, Hitachi TM-3000) (Milexia, Paris, France) was used to analyze the microstructure and morphology of the foams obtained. All samples were made into 0.5 cm^2^ (cross section). Then, a thin layer of gold–palladium was sputtered on the surface of the foams so that a better definition could be obtained.

### 2.8. Compression Strength

The samples were cut into a uniform size of 25 × 25 × 25 mm. A universal testing machine (Instron 3300, Elancourt, France) was used to test the compression strength of the foams. The direction of load was parallel to that of the foam rise under ambient conditions. The crosshead rate was fixed at 2.0 mm·min^−1^. At least three sample repeats were tested for each foam.

## 3. Results and Discussion

**Scheme 1:**
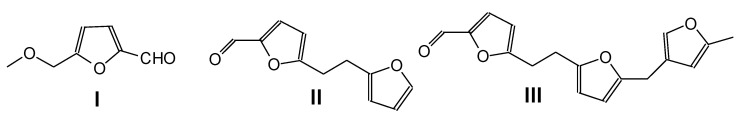
Small furanic species present in the humins used.

**Figure 1:**
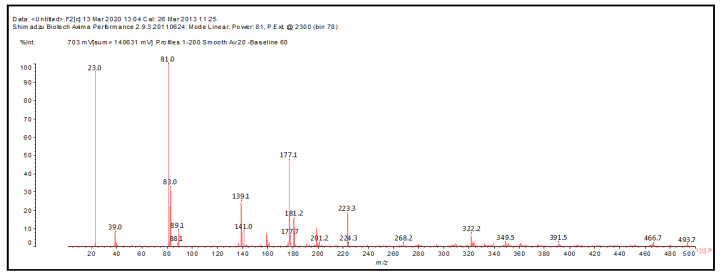
Matrix assisted laser desorption ionization time-of-flight spectrometry (MALDI ToF) spectrum of humins obtained at 180 °C from fructose, 20–500 Da range.

**Figure 2:**
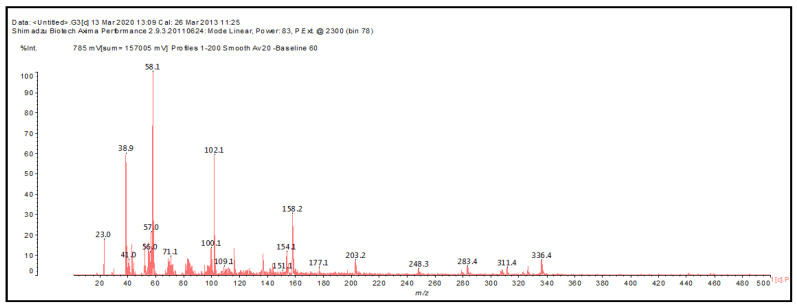
MALDI ToF spectrum of humins obtained at 150 °C from fructose, 20–500 Da range.

**Figure 3:**
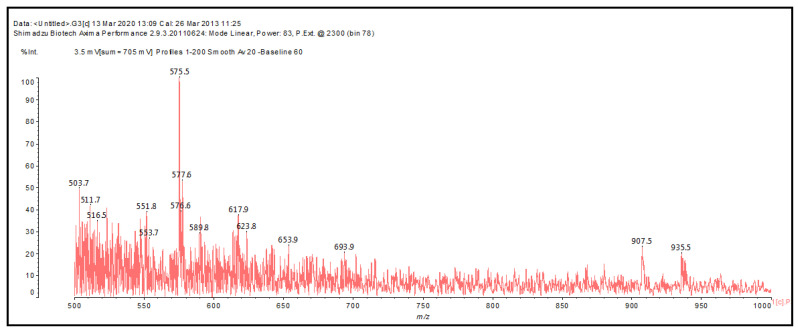
MALDI ToF spectrum of humins obtained at 150 °C from fructose, 500–1000 Da range.

**Figure 4:**
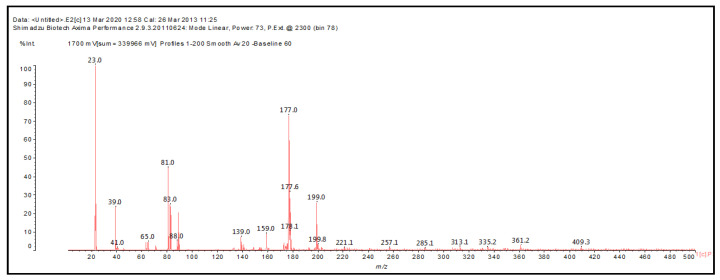
MALDI ToF spectrum of humins obtained at 180 °C from fructose, 20–500 Da range.

**Figure 5:**
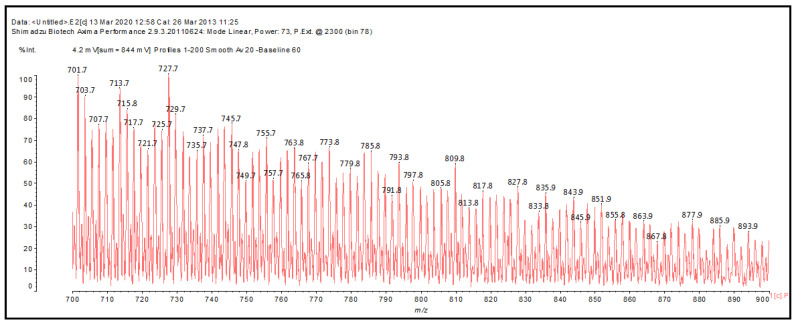
MALDI ToF spectrum of humins obtained at 180 °C from fructose, 700–900 Da range.

**Figure 6:**
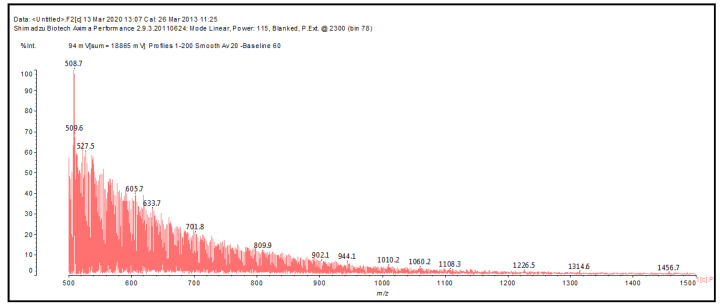
MALDI ToF spectrum of humins obtained at 180 °C from fructose, 500–1500 Da range.

Humins are known to have a polyfuranic structure [21,22,23]. However, they are a mixture of a variety of polyfuranic oligomers rather than possessing a unitary polyfuranic structure. To further clarify the types of oligomers present in the humins used for foam formulation, and to ascertain their suitability for this task, MALDI ToF analysis was carried out. Figure 1, Figure 2, Figure 3, Figure 4, Figure 5 and Figure 6 report the more significant MALDI ToF spectra (more spectra are presented in the Supplementary Material) and Table 2 reports the assigned structures that could be interpreted from the spectra. From Table 2 it can be noticed that in the mix derived from the hydrolysis of fructose either at 180 or 150 °C there are a considerable number of small furan-derived monomers, dimers and trimers, of which in particular structures of type I (139 Da Peak), II (203 Da peak) and III (284 Da peak) and their derivates are examples (Scheme 1)

**Scheme 2:**
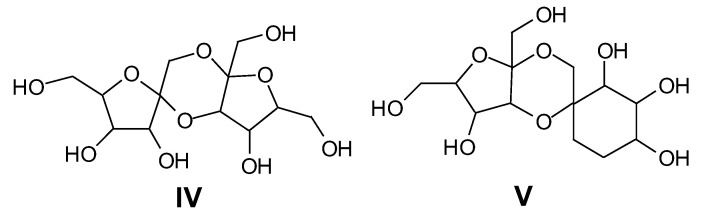
More complex small structures present in the humins used.

More complex structures such as IV and V, both corresponding to the 322 Da peak, already previously identified [22] have also been confirmed (Scheme 2).

**Scheme 3:**
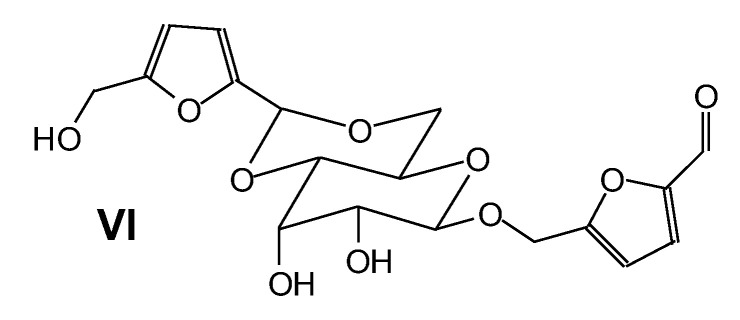
Example of furanic structures stilllinked to condensed fructose moieties present in the humins used.

Furanic structures still linked to condensed fructose moieties also appear to be present such as structure VI, assigned to the peak at 392 Da, also already previously identified [22] (Scheme 3).

**Scheme 4:**
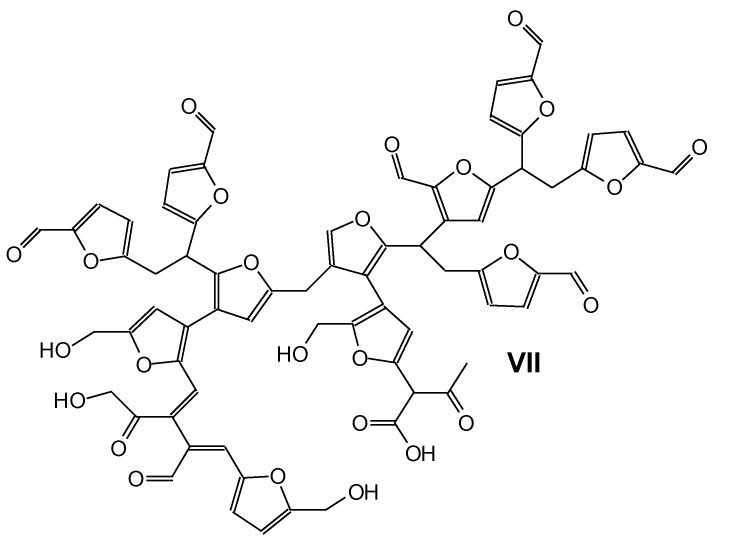
Example of a type of polyfuranic structure present in the humins used.

It is the progression from structures I, II and III that however brings to the more classical representation of the polyfuranic structure of acid-derived fructose humins, this being represented among others by the peaks at 1227, 1250 and 1351 Da (VII), such as in Scheme 4 and higher more complex oligomers. This served to control that the humins prepared presented the polyfuranic structure expected and needed to formulate the tannin–humins foams.

**Figure 7:**
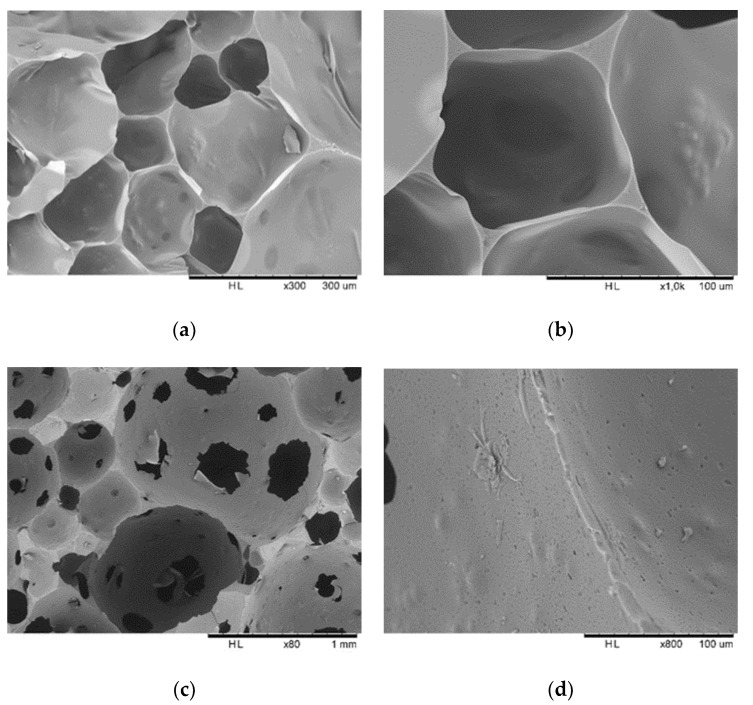
SEM of initial formulation 3 foam with magnitude of (**a**) 300× and (**b**) 10,000×, ambient temperature curing. SEM of initial formulation 4 with magnitude of (**c**) 80× and (**d**) 800×, 80 °C curing.

Two initial tannin–humins-FA formulations were prepared including two furanic foams controls. The two tannin–humins-FA were expanded and hardened one at ambient temperature (23 °C) and the other at 80 °C to see how preparation temperature influenced the morphology of the finished foams. The differences observed by scanning electron microscopy (SEM) were indeed quite major as can be seen in Figure 7. The foam prepared at ambient temperature presented a closed cells structure while the one prepared at 80 °C clearly presented an interconnected cells structure, with a number of open pores and cell walls breaks. The reason for this difference is due to the more vigorous evaporation of water at 80 °C breaking weaker cell wall sites in the structure. This does not occur at ambient temperature. This means that a foam of this type prepared at ambient temperature, once stabilized, is more suitable for thermal isolation applications, while when prepared at 80 °C it I more suitable for acoustic insulation [12].

**Figure 8:**
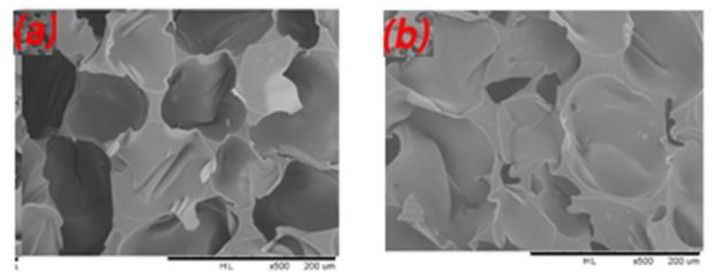
Effect on foam morphology of different proportions of water. (**a**) F-H3.7-FA6-W2.0; (**b**) F-H3.7-FA6-W1.5.

**Figure 9:**
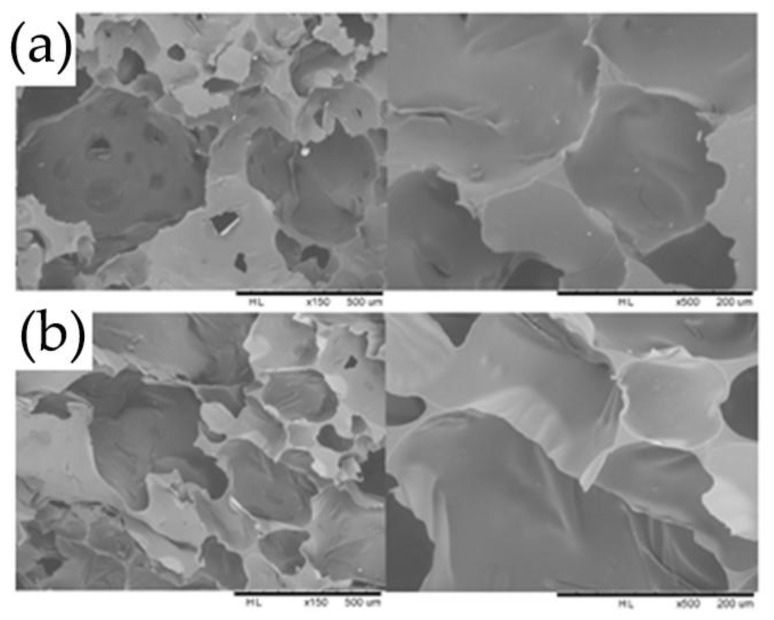
Effect on foam morphology of small difference proportions of furfuryl alcohol (FA): (**a**) F-H5.9-FA5-W1.5; (**b**) F-H5.5-FA6-W1.5.

**Figure 10:**
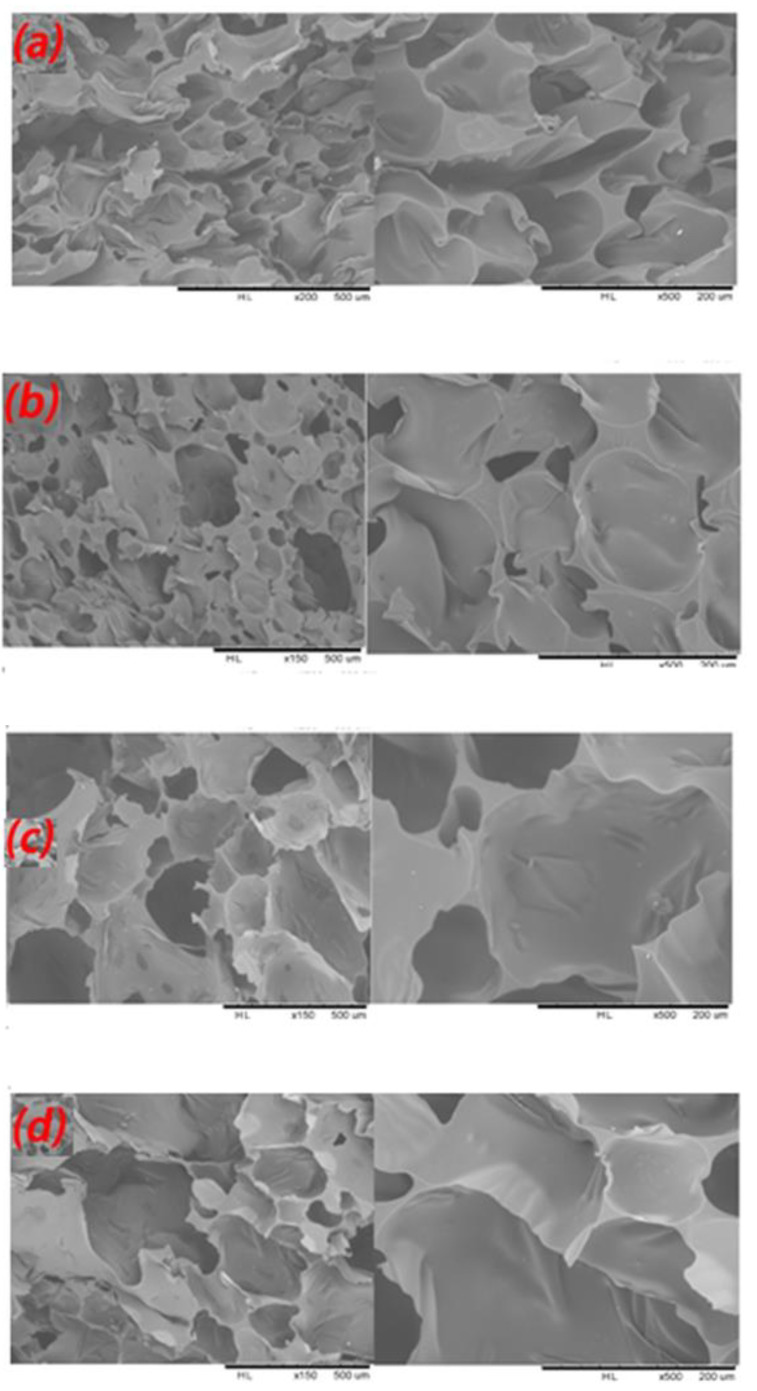
Effect on foam morphology of different proportions of humins. (**a**) F-H2.0-FA6-W1.5; (**b**) F-H3.7-FA6-W1.5; (**c**) F-H4.5-FA6-W1.5; (**d**) F-H5.9-FA6-W1.5.

**Figure 11:**
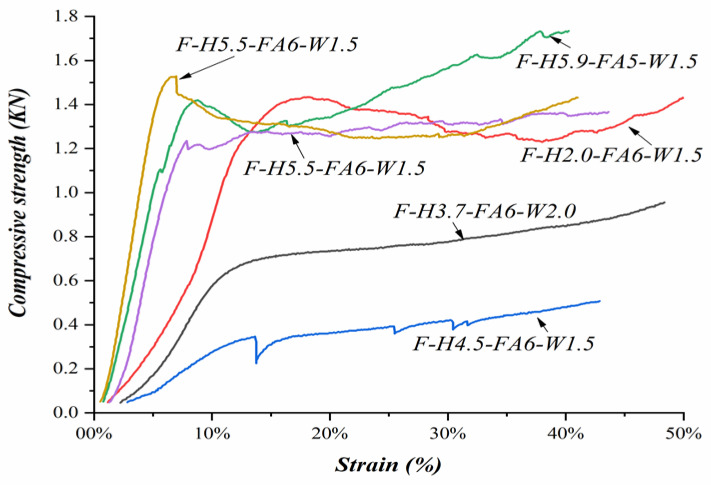
Compression strength as a function of percentage strain for the different tannin–humins foams.

The foams in Table 1 were prepared to observe the effect of the differences in formulation on foam characteristics. The influence of small differences in the proportion of the water present and of the relative differences in humins to furfuryl alcohol proportions were studied. The results obtained, shown in Table 3 and Figure 8, Figure 9, Figure 10 and Figure 11, indicated remarkable differences in behavior or structure and characteristics. SEM observation (Figure 8, Figure 9 and Figure 10) showed that: (i) a small variation in the proportion of water (Figure 8a,b) does not appear to change much the structure of the foam when cured at ambient temperature; (ii) a decrease in the proportion of furfuryl alcohol, even if not major, appears to cause some cell wall rupture (Figure 9a,b) and a more open cells structure, even if the apparent uniformity of the cell wall still appears rather uniform and strong; (iii) a variation in the relative proportions of humins to furfuryl alcohol shows instead much more apparent structural differences (Figure 10a–d): when the proportion of humins is low and FA is dominant, a closed cell structure predominates (Figure 10a). This at first transforms itself in a slightly more porous structure at the following two higher levels of humins (Figure 10b,c) proportion to return to a more closed cell structure at the highest relative proportions of humins used (Figure 10d).

These SEM observations explain the results of compression strength observed in Figure 11, and derives from the results shown in Table 3. Thus, in Figure 11 the curves of stress–strain of the different foams indicate higher compression strength as a function of strain for both the foam with the lowest and the highest proportion of humins in relation to furfuryl alcohol, and lower compression strength for the two intermediate humins proportion cases. This indicates that humins appear to be, in general, either less or slower reacting than furfuryl alcohol at ambient temperature by the time the foam is set, but that as their proportion increases to a certain critical level more of them do proportionally participate within the expansion/curing time of the foam to the reaction to contribute to its strength characteristics. This observation infers that, if moderately higher foaming/curing temperatures are used, humins would most likely participate to a greater measure to the strength of the cell walls, but would also most likely present a predominant interconnected cells structure. These conclusions are confirmed and supported by the results for overall foam density, compression strength and particularly specific compression strength in Table 2, where in effect the overall density at first progressively decreases from the H2.0 to the H3.7 and H4.5 (humins percentage on total furanic material of respectively 25%, 38% and 43%), and then progressively increases passing from the H4.5 to the H5.5 and the H5.9 (humins percentage on total furanic material of respectively 43%, 48% and 54%) all these parameters peaking for the H5.9. The compression strength and specific compression strength do follow the same trend as the density and of the strength/strain curves in Figure 11.

However, it is interesting to observe the effect of a small increase of water on the H3.7 formulation, for which the density increases slightly but leads to an impressive increase in compression strength and more noticeably of specific compression strength. This result, supported by the SEM observations, indicate that the apparent lower reactivity of humins is due, partly or even mainly, to the hindrance to its reaction consequence of its high viscosity. The reaction is then clearly diffusion-controlled, a drawback that can be partially be eliminated or decreased by adding small proportions of a solvent (in this case water). The picture that is then obtained about the use of humins for tannin–humins biofoams is that a higher proportion of humins can be used to replace in the formulation furfuryl alcohol—if small to moderate proportions of a suitable solvent, for example water, are added. An increase in temperature can also help in decreasing the humins viscosity, but while curing the reactivity/mobility problem of the material will also contribute to increase the proportion of open cells in the foam structure. It is interesting too that the compression stress at >1 MPa is rather good, and it is higher than the majority of the expanded polystyrene foams used for isolation, and higher than for the foams of humins alone [28].

**Figure 12:**
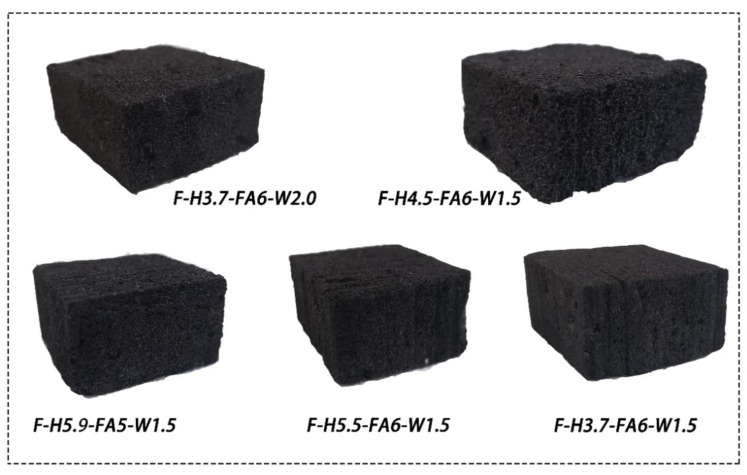
Photographic record of appearance of the experimental tannin–humins foams once cut into cubes.

An example of the appearance of the foams prepared is shown in Figure 12. The appearance of the initial exploratory foams is also shown in Figure SM3 in the Supplementary Material.

**Figure 13:**
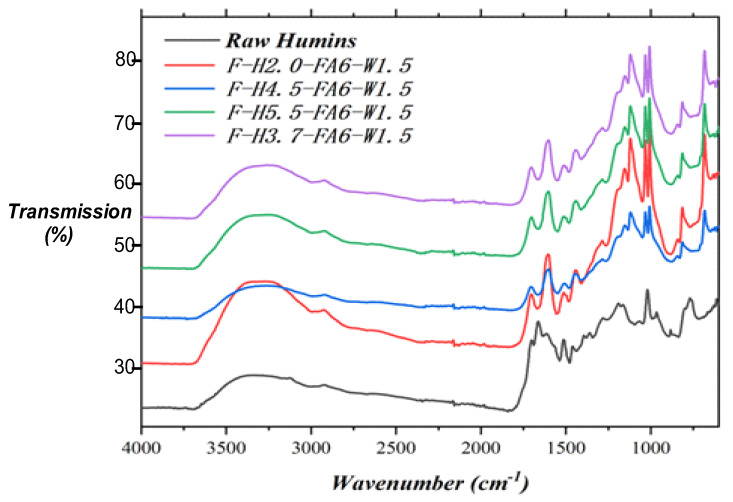
FT-IR of the different mixes of humins (H) and furfuryl alcohol (FA) used for the preparation of the tannin–humins foams.

The FT-IR of the raw humins and of the different humins-FA mixes show some interesting features as well (Figure 13). The raw humins show the peaks at 1038 cm^−1^ and the very broad peak between 3000 and 3600 cm^−1^ of hydrogen bonded alcohol groups. The three peaks at around 1700 cm^−1^ correspond to aldehyde groups under different surrounding conditions, while the 1500 cm^−1^ peak is assigned to alkane groups asymmetric stretching, and the peak at 800 cm^−1^ to alkene groups present in structures as that of the 1351 Da peak in the MALDI ToF spectra. In the experimental humins-FA mixes the peak that appear superimposed to the humins groups are the 1310 cm^−1^ and 1029 cm^−1^ assigned to the stretching of the hydrogen-bonded alcohol function of furfuryl alcohol, which appears just very near to the 1038 cm^−1^ peak of humins, The furanic structures C=C groups of both humins and furfuryl alcohol both appear at around 1200 cm^−1^ with two vicinal sharp peaks, the smaller assigned to the humins and the taller and sharper to the furfuryl alcohol.

**Scheme 5:**
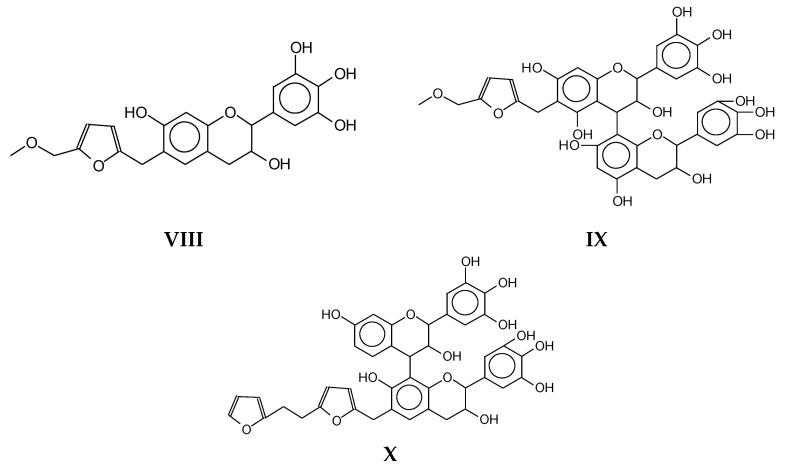
Examples of structures produced in the foams coreaction showing that flavonoid monomers and oligomers do react with simple humins structures.

**Figure 14:**
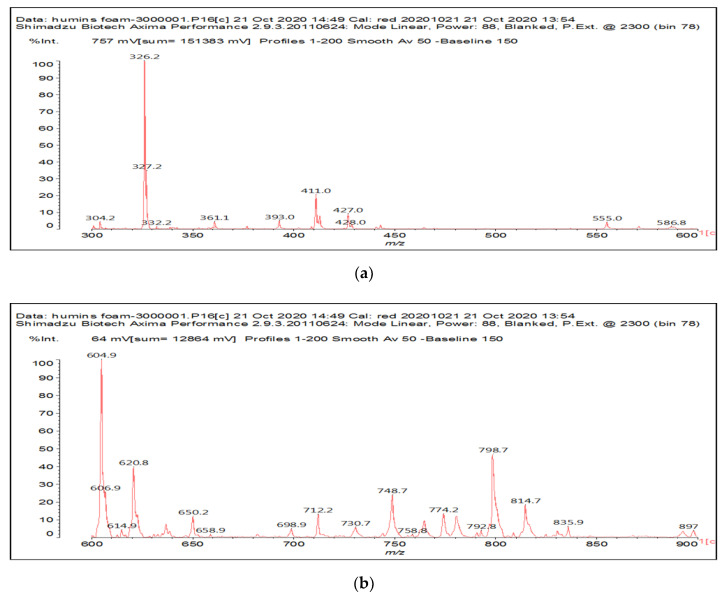

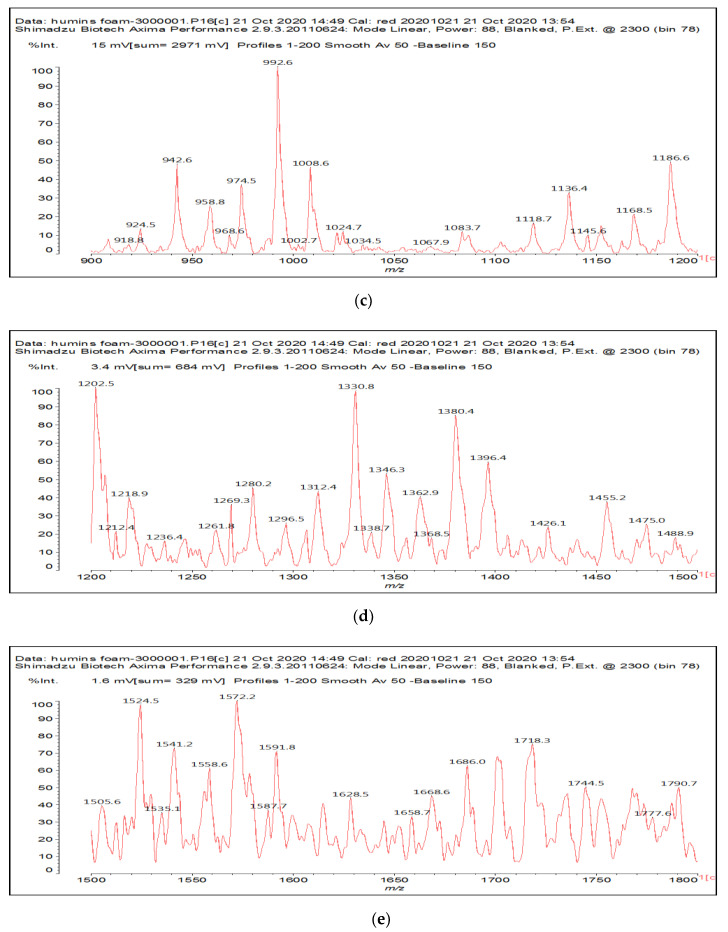

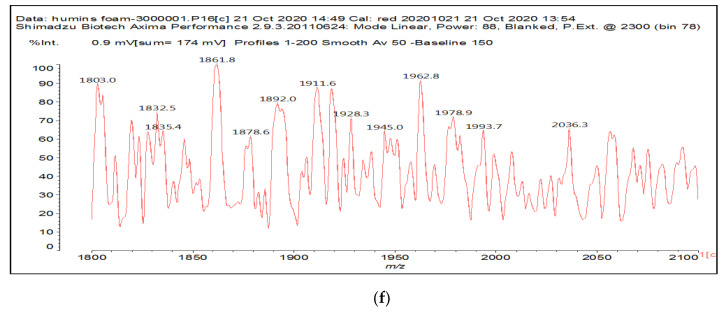
MALDI ToF spectrum of F-H4.5-FA6-W1.5 foams: (**a**) 300 to 600 Da range; (**b**) 600 to 900 Da range; (**c**) 900 to 1200 Da range; (**d**) 1200 to1500 Da range; (**e**) 1500 to 1800 Da range; (**f**) 1800 to 2100 Da range.

It is of interest to observe the results of the MALDI ToF spectra of the finished foams, to see if the three main constituents—mainly tannin extract, furfuryl alcohol and humins oligomers—have jointly reacted. The list of the compounds identified for the F-4.5 H + 6 FA + 1.5 W in Figure 14a–f is shown in Table 4. Several compounds and oligomers present in the MALDI spectra of the humins alone are also present in the MALDI spectra of the tannin–humins-FA foam, such as the ones at 621, 635, 1009, 1035, 1136 Da, and others. Additionally, present are the characteristic peaks of unreacted flavonoid monomers and dimers such as 304 and 327 Da, both for a gallocatechin monomer without and with Na+, 604 Da for a robinetinidin or catechin dimer with Na+, 607 Da for a gallocatechin dimer without Na+, 897 Da for a (gallocatechin)_2_-robinetinidin trimer without Na+, 910 Da for a gallocatechin trimer without Na+, etc. However, there are a considerable number of peaks assigned to reaction products of flavonoid monomers and dimers with humins smaller molecular weight species as well as with both humins species and furfuryl alcohol. Some of the more significative ones will be discussed here (Scheme 5).

Species of type VIII (411 Da), IX (731 and 758 Da) and X (peaks 749 and 774 Da) are obtained by reaction of humins present species with different flavonoids monomers and dimers from the tannin. The peaks at 411, 427, 731, 749, 758, 774, 792, 912, 925 Da all belong to this category of tannin–humins oligomers.

**Scheme 6:**
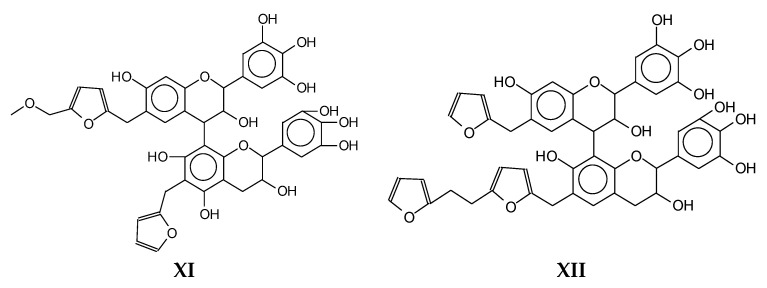
Examples of structures produced in the foams coreaction showing that flavonoid oligomers do react with both furfuryl alcohol and simple humins structures.

Mixed species showing that both furfuryl alcohol and humins species have also both reacted at the same time with flavonoid structures from the tannin are also present. Examples of these, are the co-reacted tannins-humins-furfuryl alcohol structures as the ones at 798 Da (XI), 815 Da, and 835 Da (XII) (Scheme 6).

**Scheme 7:**
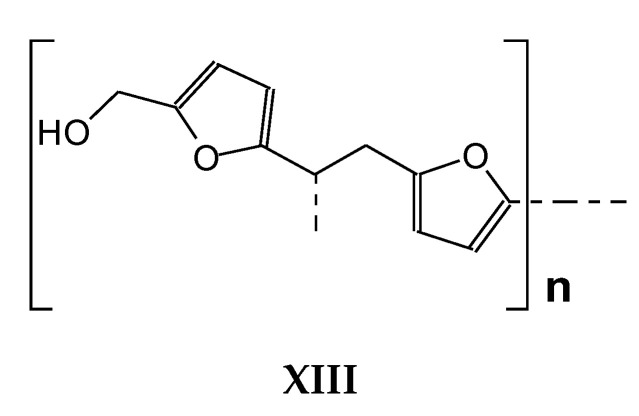
Repeating unit of one of the series of peaks observed.

There are at least two series where the successive peaks are all separated by an interval of 193–194 Da. The first of these series of oligomers belongs to the peaks at 411, 605, 799, 992, 1186, 1380, 1574, 1769, 1962 Da. The repeating motive is then a humins one, namely (XIII) (Scheme 7).

**Scheme 8:**
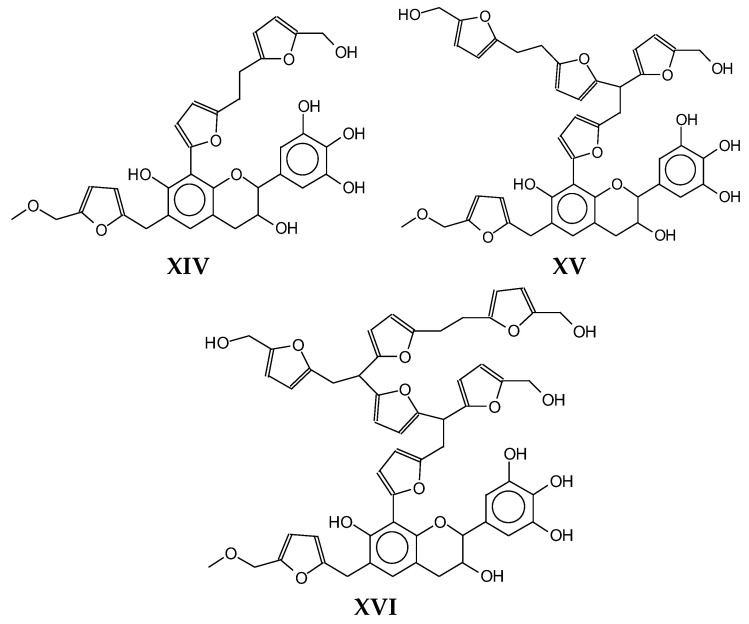
Examples of structures produced in the foams coreaction showing that flavonoid monomers and oligomers do react with complex oligomeric humins structures.

The peak at 411 Da is assigned to structure VIII above. Examples of the structures of some of the ones that follows in the series are 605 Da (XIV) and 798 Da (XV) without Na+. The species at 1008 Da (XVI) with Na+ is part of the same series. It is clear that this series of oligomers is formed by fragments of increasingly higher molecular weight humins having reacted with flavonoids (Scheme 8).

**Scheme 9:**
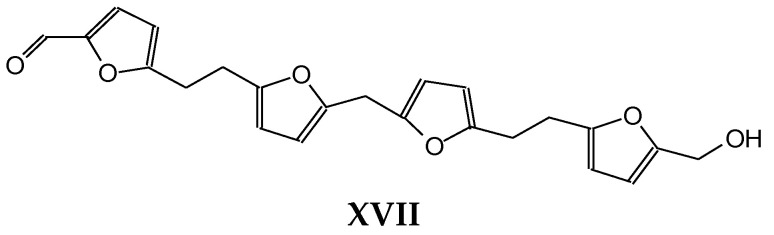
Example of reaction of simpler humins structure capable of reacting with itself to form more complex humins networks.

Equally the series 393, 587, 781, 975, 1169, 1363, 1558 Da are also separated by the same repeating unit above, this being a series of lesser peaks. This series comes exclusively from the reaction of smaller humins molecules to form the complex and sizable polyfuranic structure of the humin polymer. The first term of this series (393 Da, XVII) is shown in Scheme 9.

**Scheme 10:**
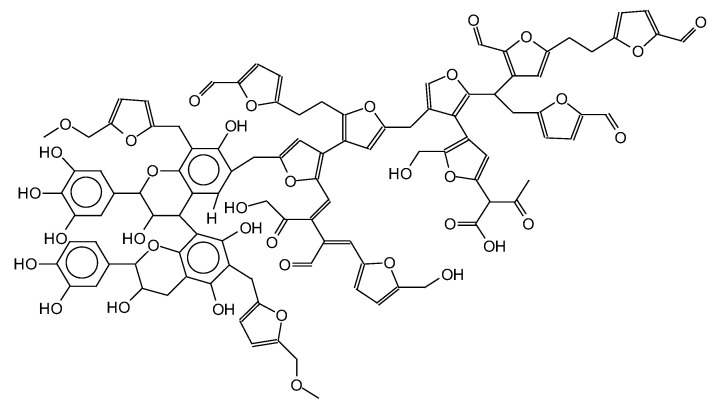
Example of higher molecular weight structure in the foams derived from the reaction of humins higher and lower molecular weight oligomers with flavonoid oligomers of the tannin.

To arrive finally to some of the higher molecular weight structures such as structure XVIII (1945 Da) where a rather sizeable humins oligomer has linked to a flavonoid dimer onto which are already linked some smaller molecular weights humins compounds (Scheme 10).

All the above indicates clearly that there is reaction between the humins oligomers and the tannin flavonoids, and principally that any humins furanic hydroxymethyl and aldehyde group, even on very high molecular weight humins oligomers, can react with the flavonoid tannin sites. The relatively high molecular weight species in the humins mix does then explain why its reaction with the tannin is slower than that of furfuryl alcohol. In the case of humins, as already remarked above, it is their bulky molecular size that determines that their reaction is heavily diffusion controlled, hence slower [33,34]. The reaction of tannin with furfuryl alcohol is particularly well studied and defined [35] and the mixed flavonoid–furfuryl alcohol-humins oligomers attest that all the three species co-react.

The above considerations explain the not ruptured morphology of the foam caused by even a very small increase in solvent. It indicates that humins foams will react better if their viscosity is lowered, either by moderately increasing their temperature, or by adding relatively low proportions of suitable solvents.

It explains why the use of slightly higher proportions of water have this effect, and why furfuryl alcohol works also as an adequate solvent to mobilize humins. When furfuryl alcohol is used not only as a reagent but also as a humins solvent, what must be considered is the relative balance of the two materials: too low a proportion of furfuryl alcohol will increase the effect of diffusion hindrance to the reaction of the humins decreasing their reaction capability, while too much of the more reactive furfuryl alcohol may limit the role of the humins in the co-reaction.

## 4. Conclusions

Ambient temperature self-blowing tannin–furanic foams, with both closed and interconnected cells, in which a great part of furfuryl alcohol has been substituted by humins, a polyfuranic material derived from the acid treatment at high temperature of fructose, have been prepared. Ambient temperature foam expansion yielded closed cells foams, while the use of a higher curing temperature (80 °C) yielded open cells foams. It must be pointed out the difference in calling some foams “open-cell” rather than “interconnected cells” foams. This difference is important, as foams having an interconnected cellular structure may combine some of the advantages of closed-cell foams (mechanical performance) and open-cell foams (absorption, acoustic applications).

They appear to present the equivalent characteristics of tannin–furanic foams prepared previously, such as self-extinguishing fire resistance, compression strength but with the advantage of a greater ease of close cells formation. The reaction of condensed tannin, furfuryl alcohol and humins has been shown to occur by the oligomers mix formed, namely tannin–humins, tannin–furfuryl alcohol and in particular tannin–humins-furfuryl alcohol, oligomer types and their structures having been determined. Humins have been shown to react well with condensed tannins, even at ambient temperature, but the reaction is slowed down by their high viscosity in relation to that of tannin with furfuryl alcohol. While even higher molecular weight humins species do appear to react with tannin, their lower rate of reaction is due to their rather high molecular weight, hence their high viscosity, causing the reaction to mainly be diffusion controlled. This feature appears to explain why even small increases in solvent proportions improve the results, and indicates the additional role of furfuryl alcohol as humins solvent and not just as a co-reagent contributing to both cross-linking and to the heat increase leading to foam expansion and its hardening.

## Figures and Tables

**Table 1:** The formulation of tannin–humins foams.

Samples	Tannin (g)	Humins (g)	FA (g)	Water (g)	p-TSA (g)	DE (g)
F-H2.0-FA6-W1.5	15.0	2.0	6.0	1.5	6.0	1.5
F-H3.7-FA6-W1.5		3.7	6.0	1.5		
F-H4.5-FA6-W1.5		4.5	6.0	1.5		
F-H5.5-FA6-W1.5		5.5	6.0	1.5		
F-H5.9-FA5-W1.5		5.9	5.0	1.5		
F-H3.7-FA6-W2.0		3.7	6.0	2.0		

**Table 2:** Assignment to relevant structures of MALDI ToF peaks of fructose laboratory derived humins.

139 Da both theoretical and experimental, no Na+
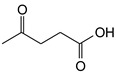
139 Da (calculated 140) no Na+
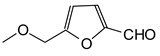
159 Da (calculated 158 Da) no Na°
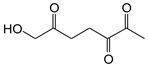
159 Da (calculated 156 Da) no Na+
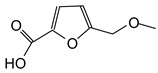
177–179 Da (Calculated 178 Da) with Na+
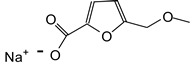
202–203 Da (Calc 203) with Na+
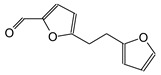
203 Da (Calc 203 Da) with Na+) [22]
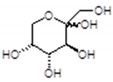
257 Da (calc 252 Da)
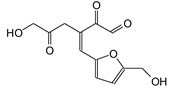
257 Da (Calc 254 Da)
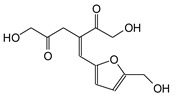
268 Da (Calculated 270 Da) no Na+, (180 °C no oxidation spectrum)
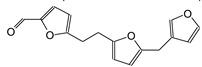
284 Da no Na+ (150 °C and 180° oxidized spectra)
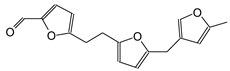
322 Da (Calculated 324 Da) no Na+ (180 °C not oxidized) [22]
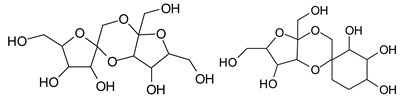
391 Da (Calculated 389 Da) with Na+, (180 °C not oxidieed)
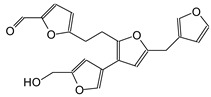
392 Da (Calculated 396 Da) (180 °C not oxidized) [22]
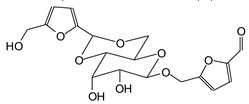
506 Da (no Na+) and 526 Da (with Na+) Calc.: 504 Da and 527 Da
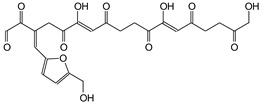
623 Da (Calculated 623 Da) with Na+ (150 °C spectrum)
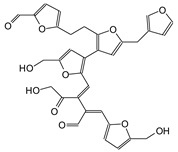
635 Da (with Na+)
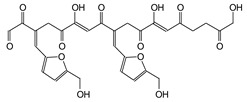
636 Da (Calculated 637 Da) with Na+, (180 °C not oxidized spectrum)
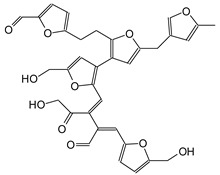
720 Da no Na+
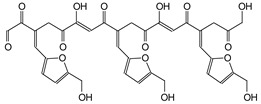
741 Da (calc 743 Da) with Na+
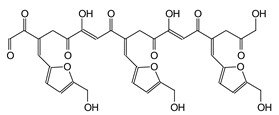
810 Da (Calculated 810 Da) without Na+ (180 °C Oxidized, 180 °C not oxidized, 150 °C)
833 Da (Calculated 833 Da) with Na+ (180 °C Oxidized, 180 °C not oxidized)
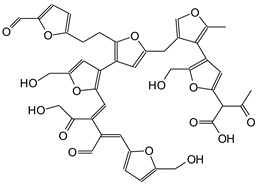
935 Da (Calculated 932 Da) without Na+ (180 °C Oxidized, 150 °C
950-952-954 Da (Calculated 955 Da) with Na+ (180 °C oxidized and not oxidized)
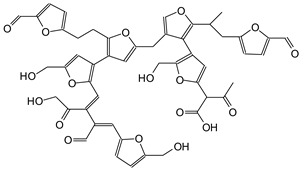
1010 Da (Calculated 1012 Da) no Na+ (180 °C not oxidized)
1033 Da (Calculated 1035 Da) with Na° (180 °C oxidized)
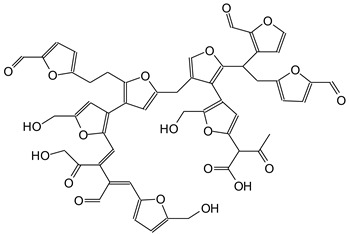
1137 Da (Calculated 1135 Da) no Na+ (180 °C oxidized)
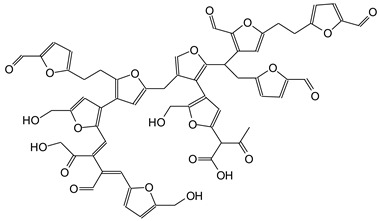
1227 Da (Calculated 1228 Da) no Na+ (180 °C not oxidized)
1250 Da (Calculated 1252 Da) with Na+ (150 °C)
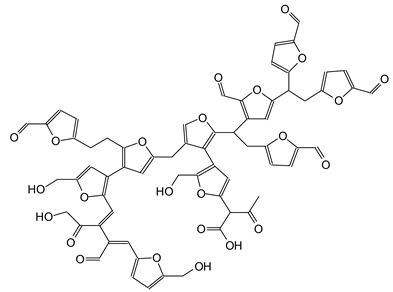
1351 Da With Na+
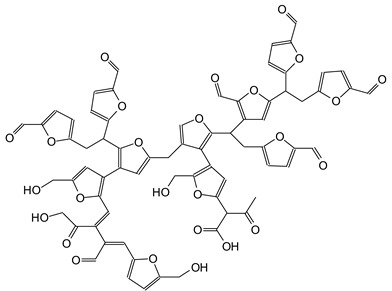

**Table 3:** Apparent density, compressive strength and specific compressive strength of tannin–humins foams.

Samples	Density (g/cm^3^)	Compressive Strength (MPa)	Specific Compressive Strength (kPa/kg∙m^−3^)
F-H2.0-FA6-W1.5	0.20	1.59	7.95
F-H3.7-FA6-W1.5	0.16	0.81	5.06
F-H4.5-FA6-W1.5	0.11	0.39	3.54
F-H5.5-FA6-W1.5	0.21	1.38	6.57
F-H5.9-FA5-W1.5	0.24	1.58	6.58
F-H3.7-FA6-W2.0	0.19	1.70	8.94

**Table 4:** MALDI ToF analysis assignments of relevant peaks of foam F-H4.5-FA6-W1.5.

304 Da Flavonoid gallocatechin, no Na+
326–327 Da Flavonoid gallocatechin with Na+
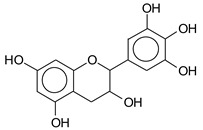
393 Da peak
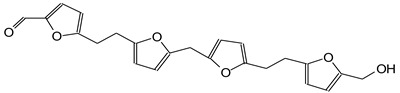
411 Da (Calculated 414 Da) no Na+
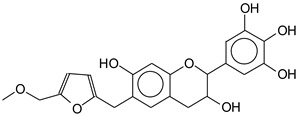
427–428 Da (Calculated 430) no Na+ reaction of a flavonoid with the humins aldehyde at 141 Da in humins only spectrum.
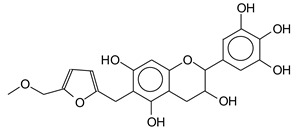
605 Da= (Calculated 605 Da) no Na+
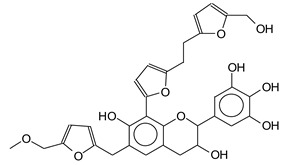
621 Da with Na+
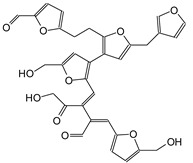
635 Da
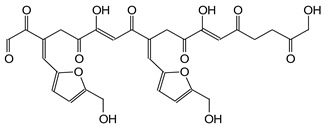
731 Da (calculated 733 Da) without Na+
758 Da (calculated 756 Da)with Na+
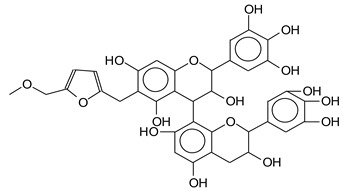
749 Da (Calculated 752 Da) no Na+, and
774 Da (Calculated 775 Da) with Na+
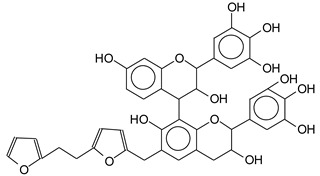
792 Da = 774 Da + 1x-OH (on A-ring of one flavonoid unit)
793 Da (Calculated 794 Da) no Na+ and 815 Da with Na+
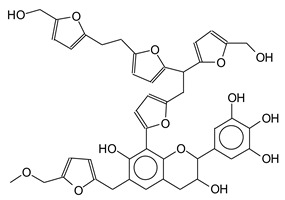
783 Da = as 798 Da but based on a fisetinidin + fisetinidin dimer
798.8 Da (Calculated 798.5 Da) no Na+. A delphinidin–robinetinidin dimer reacted with a humins low molecular weight species and with furfuryl alcohol:
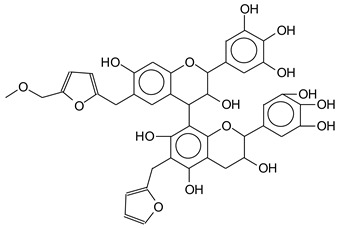
815 Da (calculated 915 Da) no Na+, reaction of flavonoid dimer with furfuryl alcohol and a 141 Da humins low molecular weight species
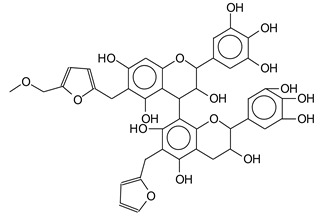
835 Da (calculated 833 Da) no Na+
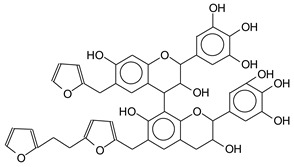
912 Da (Calculated 912) no Na+, small peak, reaction of a flavonoid dimer with polyfurfuryl alcohol and a humins compound
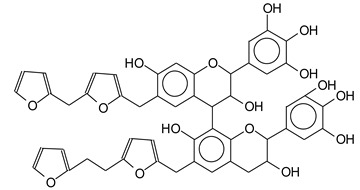
925 Da = (calculated 928 Da) = 912 + 1x-OH, no Na+
1008 Da (Calculated 1007 Da) with Na+.
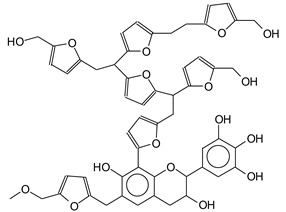
1009 Da no Na+
1035 Da (Calculated 1035 Da) with Na+ in 180 °C oxidized
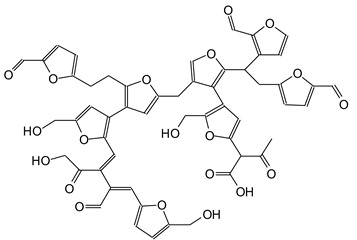
1136 Da (Calculated 1135 Da) no Na+ in 180 °C oxidized
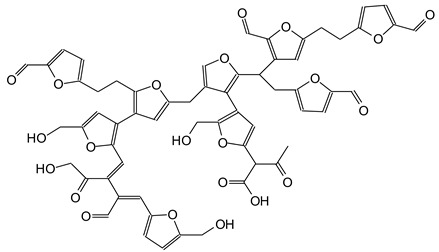
1535–1541 Da (Calc. 1538) with Na+
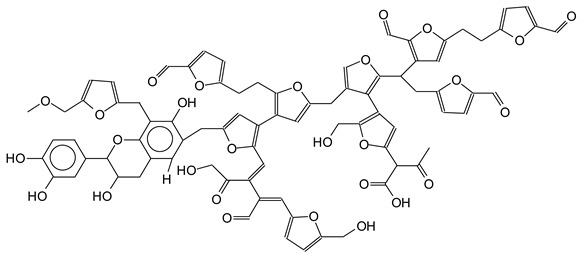
1803 Da (Calculated 1803) no Na+
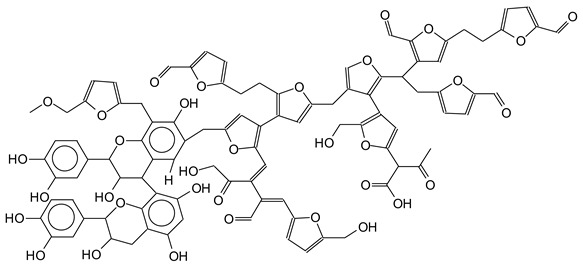
1819 Da (Calculated 1819 Da) no Na+
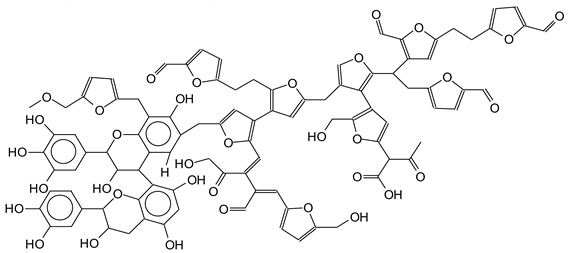
1945 Da (Calculated 1943) no Na+
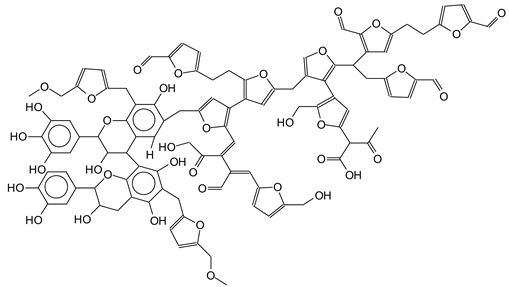

## Supplementary Materials

The following are available online at https://www.mdpi.com/2073-4360/12/11/2732/s1, Figure SM1. Title: MALDI ToF spectrum of humins obtained at 180 °C oxidized, from fructose, 1000–1500 Da range. Reported to indicate 1137 Da peak. Figure SM2. Title: MALDI ToF spectrum of humins obtained at 150 °C from fructose, 1000–1500 Da range. Reported to indicate 1250 Da peak. Figure SM3. Title: Photograph of cut section of initial tannin-humin foam formulations (a) cured at 80 °C, (b) cured at ambient temperature.

## References

[1] Pizzi A. (2019). Tannin-based biofoams-A review. J. Renew. Mater..

[2] Meikleham N., Pizzi A. (1994). Acid and alkali-setting tannin-based rigid foams. J. Appl. Polym. Sci..

[3] Tondi G., Zhao W., Pizzi A., Du G., Fierro V., Celzard A. (2009). Tannin-based rigid foams: A survey of chemical and physical properties. Bioresour. Technol..

[4] Li X., Pizzi A., Cangemi M., Fierro V., Celzard A. (2012). Flexible natural tannin-based and protein-based biosourced foams. Ind. Crops Prod..

[5] Lacoste C., Pizzi A., Basso M.C., Laborie M.-P., Celzard A. (2014). Pinus pinaster tannin/furanic foams: Part 2: Physical properties. Ind. Crops Prod..

[6] Lacoste C., Pizzi A., Basso M.C., Laborie M.P., Celzard A. (2014). *Pinus pinaster* tannin/furanic foams: Part 1. Formulation. Ind. Crops Prod..

[7] Xi X., Pizzi A., Delmotte L. (2018). Isocyanate-free Polyurethane Coatings and Adhesives from Mono- and Di-Saccharides. Polymers.

[8] Xi X., Pizzi A., Gerardin C., GDu G. (2019). Glucose-based Non-Isocyanate Polyurethane Biofoams. J. Renew. Mater..

[9] Xi X., Pizzi A., Gerardin C., Lei H., Chen X., Amirou S. (2019). Preparation and evaluation of Glucose based Non-Isocyanate polyurethane Self-blowing Rigid Foams. Polymers.

[10] Chen X., Xi X., Pizzi A., Fredon E., Zhou X., Li J., Gerardin C., Du G. (2020). Preparation and Characterization of Condensed Tannin Non-Isocyanate Polyurethane (NIPU) Rigid Foams by Ambient Temperature Blowing. Polymers.

[11] Chen X., Li J., Xi X., Pizzi A., Zhou X., Fredon E., Du G., Gerardin C. (2020). Condensed Glucose-Tannin-based NIPU BioFoams of Improved Fire Retardancy. Polym. Degrad. Stabil..

[12] Lacoste C., Basso M.C., Pizzi A., Celzard A., Ella Bang E., Gallon N., Charrier B. (2015). Pine (*P. pinaster*) and quebracho (*Schinopsis lorentzii*) tannin based foams as green acoustic absorbers. Ind. Crops Prod..

[13] Pizzi A., Tondi G., Pasch H., Celzard A. (2008). MALDI-TOF Structure determination of complex thermoset networks—Polyflavonoid tannin-furanic rigid foams. J. Appl. Polym. Sci..

[14] Tondi G., Pizzi A., Pasch H., Celzard A. (2008). Structure degradation, conservation and rearrangement in the carbonization of polyflavonoid tannin/furanic rigid foams—a MALDI-TOF investigation. Polym. Degrad. Stabil..

[15] Tondi G., Pizzi A., Olives G.R. (2008). Natural tannin-based rigid foams as insulation in wood construction. Maderas-Cienc. Tecnol..

[16] Tondi G., Pizzi A. (2009). Tannin based rigid foams: Characterisation and modification. Ind. Crops Prod..

[17] Basso M.C., Pizzi A., Al-Marzouki F., Abdalla S. (2016). Horticultural/hydroponics and floral foams from tannins. Ind. Crops Prod..

[18] Basso M.C., Pizzi A., Celzard A. (2013). Influence of formulation on the dynamics of preparation of tannin based foam. Ind. Crops Prod..

[19] Basso M.C., Pizzi A., Celzard A. (2013). Dynamic monitoring of tannin foams preparation: Surfactant effects. BioResources.

[20] Basso M.C., Lagel M.C., Pizzi A., Celzard A., Abdalla S. (2015). First tools for tannin-furanic foams design. BioResources.

[21] Sangregorio A., Guigo N., van der Waal J.C., Sbirrazzuoli N. (2018). Humins from Biorefineries as Thermoreactive Macromolecular Systems. ChemSusChem.

[22] Sangregorio A. (2019). Valorisation of Biorefinery-Derived Humins: Towards the Development of Sustainable Thermosets and Composites. Ph.D. Thesis.

[23] Van Zandvoort I., Wang Y., Rasrendra C.B., van Eck E.R.H., Bruijnincx P.C.A., Heeres H.J., Weckhuysen B.M. (2013). Formation, Molecular Structure, and Morphology of Humins in Biomass Conversion: Influence of Feedstock and Processing Conditions. ChemSusChem.

[24] Van Zandvoort I., Koers E.J., Weingarth M., Bruijnincx P.C.A., Baldus M., Weckhuysen B.M. (2015). Structural characterization of ^13^C-enriched humins and alkali-treated ^13^C humins by 2D solid-state NMR. Green Chem..

[25] Hoang T.M.C., van Eck E.R.H., Bula W.P., JGardeniers J.G.E., Lefferts L., Seshan K. (2015). Humin based by-products from biomass processing as a potential carbonaceous source for synthesis gas production. Green Chem..

[26] Sangregorio A., Guigo N., de Jong E., Sbirrazzuoli N. (2019). Kinetics and Chemorheological Analysis of Cross-Linking Reactions in Humins. Polymers.

[27] Sangregorio A., Muralidhara A., Guigo N., Marlair G., Angelici C., Thygesen L.G., de Jong E., Sbirrazzuoli N. (2020). Humins based resin for wood modification and properties improvement. Green Chem..

[28] Mija A.C., de Jong E., van der Waal J.C., van Klink G.P.M. (2020). Humins-Containing Foam. U.S. Patent.

[29] Licsandru E., Mija A.C. (2019). From biorefinery by-product to bioresins. Thermosets based on humins and epoxidized linseed oil. Cell. Chem. Technol..

[30] Cornillet C., Labat G., Gaillard J.-M. (2012). FCBA, Analyse des Cycles de Vie (LCA), Presentation project BEMA, 16 February 2012. Restitution du programme Bois Eco Matériaux Aquitaine (BEMA).

[31] Arias A., González-García S., Feijoo G., Moreira M.T. (2020). Tannin-based bio-adhesives for the wood panel industry as an environmentally friendly alternative to petrochemical resins. J. Ind. Ecol..

[32] Xi X., Pizzi A., Lei H., Du G., Zhou X., Lin Y. (2020). Characterization and preparation of furanic-glyoxal foams. Polymers.

[33] Goodner M.D., DeSimone J.M., Kiserow D.J., Roberts G.W. (2000). An Equilibrium Model for Diffusion-Limited Solid-State Polycondensation. Ind. Eng. Chem. Res..

[34] Aronhime M.T., Gillham J.K., Dušek K. (1986). Time-temperature-transformation (TTT) cure diagram of thermosetting polymeric systems. Epoxy Resins and Composites III. Advances in Polymer Science.

[35] Abdullah U.H.B., Pizzi A. (2013). Tannin-Furfuryl alcohol wood panel adhesives without formaldehyde. Eur. J. Wood Prod..

