# Supplementary material for: Ambient Temperature Self-Blowing Tannin-Humins Biofoams
Source: Polymers (Basel). 2020 Nov 17;12(11):2732. doi: 10.3390/polym12112732 (PMC7698803; doi:10.3390/polym12112732)
Supplement: Supplementary file 1 [file polymers-12-02732-s001.pdf]

SUPPLEMENTARY MATERIAL

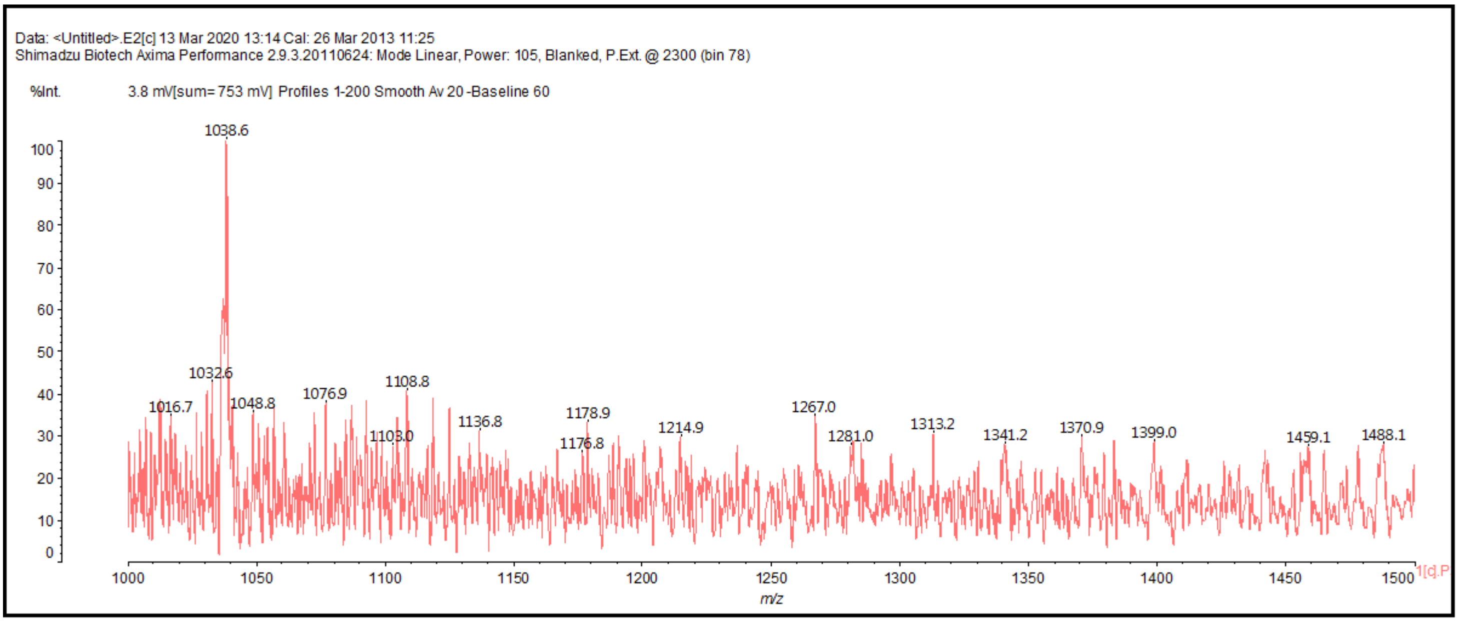

Figure SM1. MALDI ToF spectrum of humins obtained at 180°C oxidized, from fructose, 1000 Da-1500 Da range. Reported to indicate 1137 Da peak.

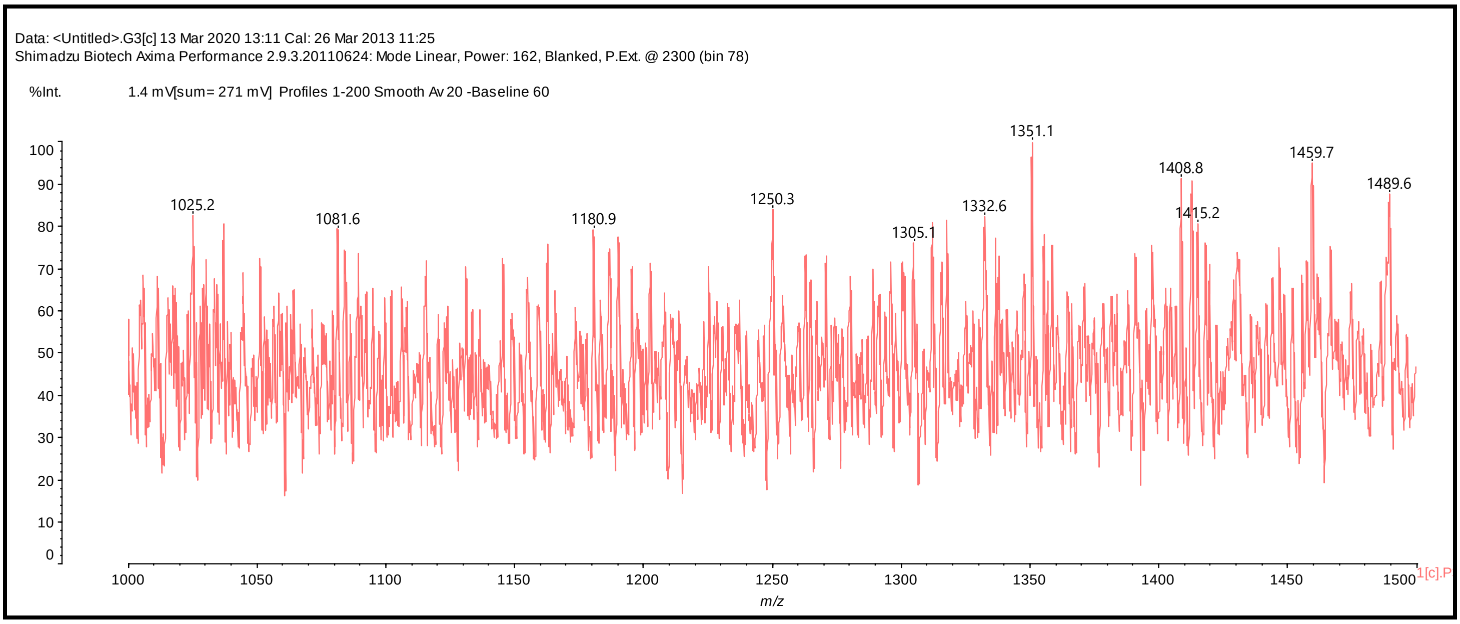

Figure SM2. MALDI ToF spectrum of humins obtained at 150°C from fructose, 1000 Da-1500 Da range. Reported to indicate 1250 Da peak.

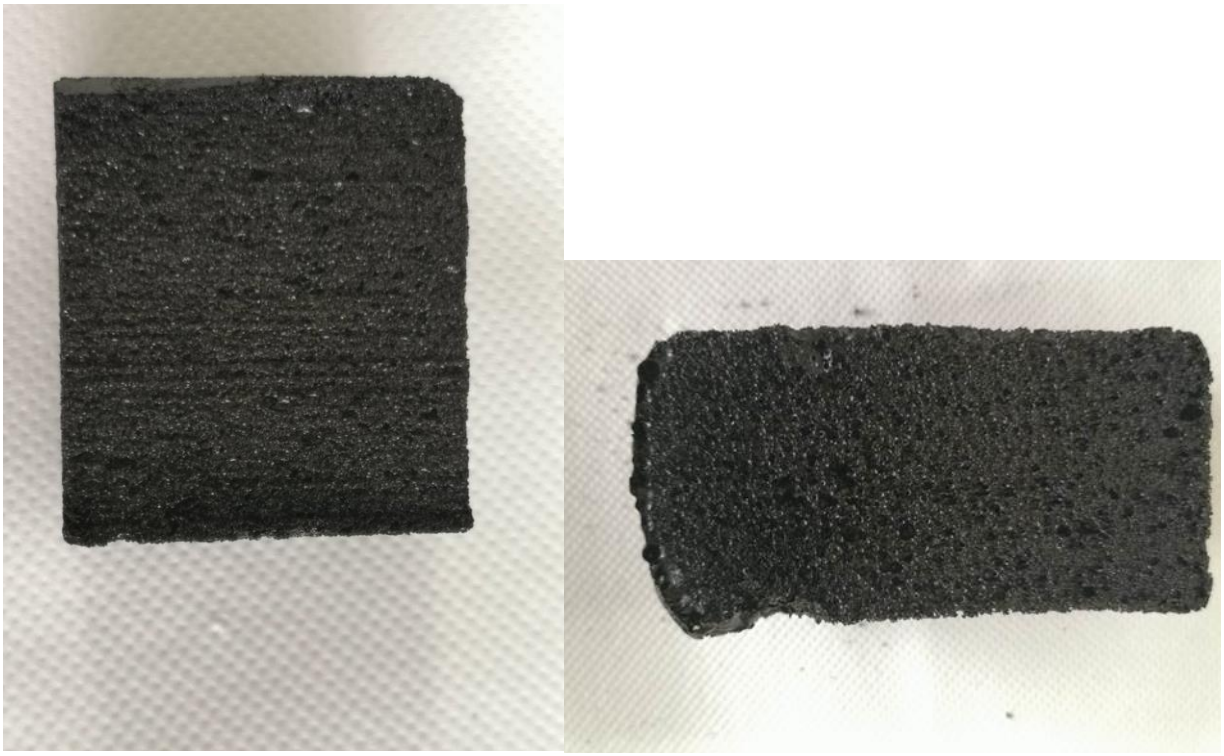

Figure SM3. Photograph of initial tannin-humin foam formulations (a) cured at 80°C, (b) cured at ambient temperature.
